# Towards best practice in developing motor skills: a systematic review on spacing in VR simulator-based psychomotor training for surgical novices

**DOI:** 10.1186/s12909-023-04046-1

**Published:** 2023-03-13

**Authors:** Jan Torge Fahl, Robbert Duvivier, Laurens Reinke, Jean-Pierre E. N. Pierie, Johanna Schönrock-Adema

**Affiliations:** 1 University of Groningen, University Medical Center Groningen, Groningen, The Netherlands; 2 Wenckebach Institute for Education and Training, Simulation Center, University Medical Center Groningen, Groningen, The Netherlands; 3 Department of Surgery, Medical Center Leeuwarden, Leeuwarden, Netherlands; 4 Hanze University of Applied Sciences, Groningen, The Netherlands

**Keywords:** Spaced training, Virtual reality, Medical education, Psychomotor skill, Surgical training

## Abstract

**Objective:**

Repeated practice, or spacing, can improve various types of skill acquisition. Similarly, virtual reality (VR) simulators have demonstrated their effectiveness in fostering surgical skill acquisition and provide a promising, realistic environment for spaced training. To explore how spacing impacts VR simulator-based acquisition of surgical psychomotor skills, we performed a systematic literature review.

**Methods:**

We systematically searched the databases PubMed, PsycINFO, Psychology and Behavioral Sciences Collection, ERIC and CINAHL for studies investigating the influence of spacing on the effectiveness of VR simulator training focused on psychomotor skill acquisition in healthcare professionals. We assessed the quality of all included studies using the Medical Education Research Study Quality Instrument (MERSQI) and the risk of bias using the Cochrane Collaboration’s risk of bias assessment tool. We extracted and aggregated qualitative data regarding spacing interval, psychomotor task performance and several other performance metrics.

**Results:**

The searches yielded 1662 unique publications. After screening the titles and abstracts, 53 publications were retained for full text screening and 7 met the inclusion criteria. Spaced training resulted in better performance scores and faster skill acquisition when compared to control groups with a single day (massed) training session. Spacing across consecutive days seemed more effective than shorter or longer spacing intervals. However, the included studies were too heterogeneous in terms of spacing interval, obtained performance metrics and psychomotor skills analysed to allow for a meta-analysis to substantiate our outcomes.

**Conclusion:**

Spacing in VR simulator-based surgical training improved skill acquisition when compared to massed training. The overall number and quality of available studies were only moderate, limiting the validity and generalizability of our findings.

**Supplementary Information:**

The online version contains supplementary material available at 10.1186/s12909-023-04046-1.

## Background

When acquiring new psychomotor skills, surgical novices need to train repeatedly. VR-simulators can provide a safe learning environment and allow the learner to repeatedly practice movements and skills without compromising patient safety. In addition, research has shown that training is more effective if spaced across multiple sessions than a single, long training session. Thus, both spaced learning and VR-simulators may have positive effects on learning and skill acquisition. However, it remains unclear how a spaced training schedule and VR based psychomotor training can effectively be linked together for further enhanced psychomotor skill acquisition.

Therefore, this systematic review aspires to confirm the effectiveness of spaced training when using a VR-simulator and identify an optimal spacing interval to acquire new surgical psychomotor skills.

## Introduction

Repeated practice is essential to reach surgical proficiency, even for the most gifted individuals [1]. It is widely acknowledged that as much as 10 years of intense, goal-directed practice are needed to attain surgical mastery [2]. VR simulators may support the process of reaching proficiency by allowing repeated practice, while focusing on single steps in a complex chain of varying tasks. In addition, VR simulators can render relevant anatomical structures with a degree of realism that conventional analogue trainers cannot provide [3]. Besides, VR simulators promote efficient learning as they facilitate self-directed learning through objective and immediate feedback on performance and performance statistics [2, 4].

Several studies provided support for the effectiveness of VR simulator-based psychomotor skill training in clinical education [4–6]. A 2011 meta-analysis showed that teaching medical skills using simulation was superior to traditional clinical education [7]. For example, learners who had completed VR laparoscopic cholecystectomy training progressed 29% faster and were significantly less likely to fail a gallbladder dissection in the real operating room than learners who had completed standard programmatic training [8]. In the approach of conventional medical curricula – known as the Halstedian approach – internship-based exposure to patients and clinical experience are key-determinants for novice doctors to become competent clinical practitioners [3, 9]. However, this approach is fraught with problems: simply exposing trainees to clinical practice does not guarantee proficiency and a doctor’s lack of experience is a known risk factor for adverse surgical outcomes [10]. When using a simulator, the early learning phase with its high risk of errors is moved from the operating room to a safe learning environment, in which novice doctors can repeatedly practice movements and skills without compromising patient safety [1, 3, 10, 11]. VR training has been shown to be at least as effective as alternative training methods such as video trainers, implying that VR training can supplement traditional laparoscopic training [12]. Similarly, a 2020 meta-analysis demonstrated that orthopaedic VR simulators improved task efficiency and overall performance in joint arthroscopy [13].

Apart from the positive effects of VR simulators on training effectiveness, several studies suggested that spaced training schedules could be a promising approach to further increase surgical training effectiveness [3, 4, 12, 14, 15]. Versteeg et al. defined spaced learning as “educational encounters that are devoted to the same material, distributed over a number of periods separated by an interstudy interval […]” [16]. Extensive singular training sessions (i.e., massed training) bear the risk that the learner’s ability to concentrate diminishes after longer practice periods, and that detrimental factors like fatigue and boredom reduce the effectiveness of training [2, 17]. This effect is known as reactive inhibition and has been demonstrated to negatively impact psychomotor skills learning [18–21]. A short rest of just 5 min can already drastically attenuate the effect of reactive inhibition [21]. Spacing training sessions over consecutive days, weeks or even months instead of multi-hour training sessions or weekend seminars may further minimize the effect of reactive inhibition and, therefore, improve skill acquisition [3, 18, 21, 22]. Research showed that spaced training with breaks offering the opportunity to sleep (i.e., lying in a bed, reduced activity, reduction of visual-auditory input) were more effective than conventional massed training [17]. An alternative explanation for the superior results of spacing is that there is simply more time for psychomotor skills to be cognitively consolidated between training sessions [3]. Regardless of whether rest periods enhance skill acquisition through cognitive consolidation or by reducing reactive inhibition, trainees following spaced training schedules outperformed trainees following massed training despite identical training loads [15, 18]. Although the benefits of spacing on learning in general were already discovered in 1885 and despite scientific evidence for positive effects of spacing in clinical training [18, 23–25], spaced training is only slowly being incorporated into medical curricula, possibly because of inconclusive findings regarding the optimal spacing interval [11, 26, 27].

Despite the positive effects of spaced training and the potential of VR-based psychomotor skill training to deliver spaced training without compromising patient care, to our knowledge, no systematic review has been performed yet to identify the optimal spacing interval in VR-based psychomotor skill training. VR-simulators function fundamentally differently from other simulators and are highly suitable for spaced training. They are becoming increasingly important in clinical training due to the growing use of robot-assisted surgery and a progressive digitalisation of the clinical training. At the same time, findings on the optimal spacing interval are inconclusive, which underlines the significance of focusing our systematic review on spaced VR-simulator training. Therefore, we conducted a systematic review to investigate the impact of temporal spacing of VR simulator training on surgical psychomotor skill acquisition. Further insight in the effectiveness of spacing may advance the development and implementation of VR-based psychomotor skill training and, therefore, improve trainees’ proficiency and patient safety. Our research questions were:

1. Is spaced VR simulator training aimed at acquiring surgical psychomotor skills superior to massed training?
2. What is the optimal spacing interval for VR simulator training aimed at acquiring surgical psychomotor skills?

## Methods

We performed this systematic review in accordance with the PRISMA statement checklist [28].

### Search strategy

We designed our search string to be highly sensitive rather than specific, since we intended to obtain as many articles as possible about VR simulator-based surgical psychomotor skill training. We identified five important elements in our research question: *evaluation*, *distributed learning*, *psychomotor skill*, *medical education,* and *simulation*. After an initial broad explorative search, we screened several publications to identify relevant terminology. For each element, we collected synonyms and commonly used free text search terms. We also took various ways of spelling into consideration. Additionally, we added thesaurus terms –like MeSH-terms in PubMed– for the elements *skill evaluation* and *medical education*. For the elements *distributed learning*, *psychomotor skill* and *simulation*, we limited our search to the fields title and abstract to find articles that specifically focused on surgical psychomotor skill acquisition using VR simulator-based training. All search terms within one element were combined with the Boolean operator “OR”, while we linked the five elements with an “AND” operator. On the 24th of June 2020, we systematically searched five online databases: PubMed, PsycINFO, Psychology and Behavioral Sciences Collection, ERIC and CINAHL. During data analysis and manuscript writing, we performed auxiliary searches on the 20th of November 2020 and the 15th of January 2022 to retrieve recently published articles. However, we did not retrieve any relevant new articles to be included in this review. The full search string is provided in Supplementary file 1. We screened the reference lists of and citations to the included articles (snowballing method) to identify additional relevant articles.

### Inclusion and exclusion criteria

Inclusion and exclusion criteria were established before the start of the reviewing process. All included articles had to meet the following inclusion criteria:

1. **Language:** English, Dutch or German;
2. **Population:** The study focused on healthcare professionals and aspiring medical personnel;
3. **Intervention:** The study used a VR simulator with a spaced training program;
4. **Outcome:** The study focused on psychomotor skill acquisition;
5. **Availability:** The article was readily accessible through the university library or online databases;
6. **Publication type:** Peer-reviewed, published primary studies.

Studies which did not meet the inclusion criteria were excluded.

### Study selection

We imported the results from our database searches into EndNote X9 and exported them to Rayyan, a web-based application for systematic reviews meant to facilitate both the research process and collaboration within the research team [29, 30]. After removing duplicates, the first author (JTF) and a peer researcher (TG) screened the abstracts and titles of a subset of 5% of the articles, as previously described by Versteeg et al. [16], to ensure consistent application of inclusion criteria. According to best practice guidelines for abstract screening, at least 30 abstracts were to be screened to avoid false judgement and minimize the risk of bias [31]. Any differences in rating during the initial calibration exercise were resolved by open discussion. Given the high interrater agreement (≥90%), JTF then independently screened the titles and abstracts of the remaining articles. All articles that met the inclusion criteria were marked as potentially relevant. A rigorous methodology was applied during the title and abstract screening phase with articles being leniently marked as “potentially relevant” in case their relevance was not yet clear. Subsequently, both researchers individually screened the full texts of all potentially relevant articles. Disagreements were resolved by discussion or brought to the larger research team for individual full text screening, discussion, and inclusion decisions.

### Assessment of study quality

We assessed the quality of all studies using the Medical Education Research Study Quality Instrument (MERSQI) [32], which involved (a) scoring of 10 items reflecting 6 domains of study quality: “Study design” [possible score 1-3 points], “sampling” [0.5-3 points], “type of data” [1-3 points], “validity of evidence for evaluation instrument scores” [0-3 points], “data analysis” [0-3], and “outcome” [1-3 points] and (b) combining the six domain scores into an overall score [32]. The maximum domain score was 3 and the maximum overall score was 18.

To assess the quality and bias of the included studies, we used the Cochrane Collaboration’s risk of bias assessment tool [33]. Two authors (JTF and LR) independently rated the articles based on random sequence generation (selection bias), allocation concealment (selection bias), blinding of participants and personnel (performance bias), blinding of outcome assessment (detection bias), incomplete outcome data (attrition bias), selective reporting (reporting bias), and other bias. For each study, the risk of bias was rated as “high”, “low” or “unclear”.

### Data extraction

JTF extracted all data regarding study aim, country, study design, number of participants, participants profile, simulator used (type, brand, country), training schedules, metrics assessed, psychomotor task assessed, spacing interval, and outcomes from the included studies. A peer researcher (TG) randomly verified the extracted information.

## Results

**Fig. 1:**
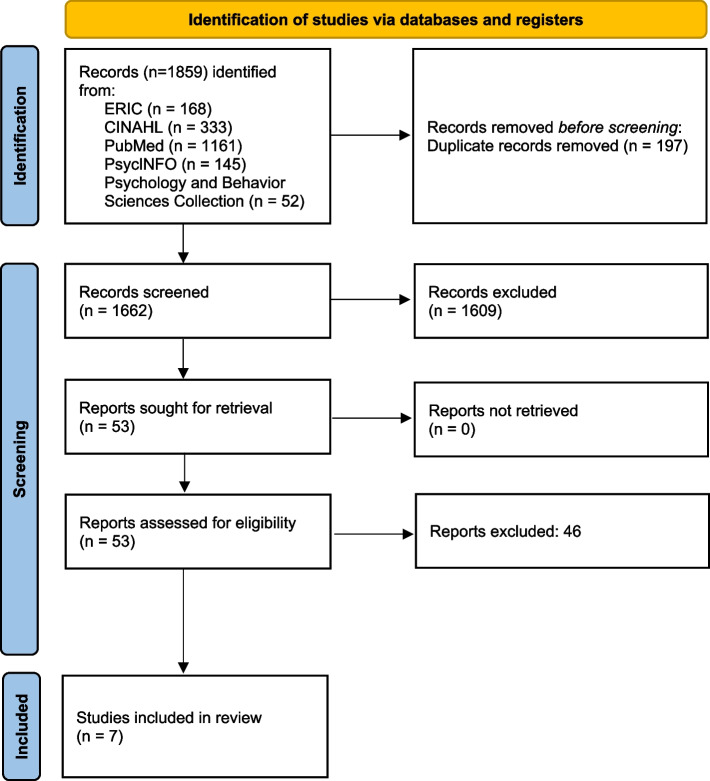
PRISMA flow diagram visualising the identification, screening and inclusion of studies in this review

Our search yielded 1859 records across five databases, see Fig. 1. After removing 197 duplicates, 1662 records remained for title and abstract screening. The abstract and title screening of 98 records (5% subset screening for calibration purposes) yielded a high inter-rater agreement of 98% (identical rating for 96 out of 98 records). Given the high inter-rater agreement, JTF then continued independently with the title and abstract screening. In total, 53 of the 1662 records were considered potentially relevant and retained for full-text screening. Of the 53 studies, 7 met the inclusion criteria. The remaining 46 records were excluded because of a variety of reasons, mainly because the studies did not use VR simulators, were published in a journal that was not peer-reviewed, or were no primary studies investigating the effect of spacing on skills acquisition. Screening of the reference lists and the citations of the included articles yielded no additional eligible publications. The full PRISMA flow diagram is available in Supplementary file 2.

Quality assessment using the MERSQI yielded a mean score of 14.14 (range 14-16). “Study design*”* was rated average to high because, in all studies, participants were randomly assigned to at least two independent groups. For the domain “sampling”, maximum scores were given for “response rate” since in all studies, participants were enrolled in a training program, which naturally yields a high attendance rate. “Number of institutions studied”, however, was rated average to low, because in all but one study participants were recruited from a single institution. Similarly, for the domain “validity of evidence for evaluation of instrument scores”, “internal structure” could not be rated for most of the studies. Besides, we did not identify any considerable statistical errors or insufficient transparency. Considering that the data analysis of all studies exceeded the level of descriptive analysis, we gave all studies the maximum score of two points for “data analysis sophistication” and a full score for “data analysis appropriateness”. Regarding the quality of “outcomes”, all studies focused on skill acquisition, rather than patient health outcomes, resulting in a low to average score of 1.5. The full MERSQI rating is available in Supplementary file 3.

The risk of bias assessment yielded an overall low risk of bias. All ratings can be found in Table 1 [33]. All included studies were predominantly rated with a low risk of methodological selection, performance, detection, or attrition bias. Andersen et al. and Kang et al. were rated with a high risk for allocation concealment, since the allocation of participants to spaced and massed training conditions was insufficiently described, participants were recruited from a single institution or not randomized [34, 35]. In general, the study design of the included studies did not allow for blinding of participants for study condition or performance metrics (e.g., time to completion). We did not expect an effect from awareness of allocation to spaced or massed training conditions on performance, especially since there were no differences between the conditions in amount of training, kind of training and in feedback from the VR simulators or instructors. Since the conditions differed only in terms of spacing, we considered the risk of performance bias to be low. Apart from that, the lack of blinding applied to all studies included in our review, which means that our judgement on this aspect does not have an impact on the ranking of the studies regarding risk of performance bias. The detailed risk assessment can be found in Supplementary file 4.

**Table 1:** Outcomes of risk of bias assessment of all included studies

Study	Mackay et al. 2002 [19]	Andersen et al. 2015 [34]	Kang et al. 2015 [35]	Bjerrum et al. 2016 [36]	Güldner et al. 2017 [37]	Gallagher et al. 2012 [38]	Verdaasdonk et al. 2006 [39]
Random sequence generation (selection bias)	Low risk	High risk	High risk	Low risk	Low risk	Low risk	Low risk
Allocation concealment (selection bias)	Low risk	High risk	High risk	Low risk	Low risk	Low risk	Low risk
Blinding of participants and personnel (performance bias)	Low risk	Low risk	Low risk	Low risk	Low risk	Low risk	Low risk
Blinding of outcome assessment (detection bias)	Unclear risk	Low risk	Low risk	Low risk	Low risk	Low risk	Low risk
Incomplete outcome data (attrition bias)	Low risk	High risk	Low risk	Low risk	Low risk	Low risk	Low risk
Selective reporting (reporting bias)	Low risk	Unclear risk	Low risk	Low risk	Low risk	Low risk	Low risk
Other bias	Low risk	Low risk	Low risk	Unclear risk	Low risk	Low risk	Low risk

### General characteristics

All studies included in our systematic review were published between 2002 and 2017. A variety of VR simulators were used to assess different surgical metrics and tasks, such as suturing or laparoscopic transfer-place tasks. The number of participants in the studies varied between 20 and 41. The participants were either medical students or novice residents and had no previous experience with the used simulators. Performance measurement was predominantly done during the training, with some studies conducting a pre-test and/or a post-training follow-up test. The training schedules varied considerably: In two studies, a daily training was compared to a massed control, in two studies a weekly training was compared to a massed control and in two studies a one-day training using a spaced schedule was compared to a weekly spaced training. The study design of Mackay et al. differed from the other six studies, because they compared a massed training to two spaced training schedules with a noticeably shorter spacing interval (2.5 minutes). One of the two spaced training schedules also had a shorter total training time (15 minutes instead of 20 minutes) [19]. Overall, in the included studies, the spacing intervals varied from 2.5 minutes to 7 days [19, 35–37]. All extracted information, including study design, profiles of participants, metrics assessed and outcomes are provided in full detail in Table 2.

**Table 2:** Key information and results of all included studies

Study	Aim	Country	Study design	N subjects	Profile of participants	Simulator (type, brand)	Groups / schedules	Total training duration	Spacing interval	Task	Metrics assessed	Testing regime:	Outcome ( p ≤ .05 is considered significant)
Mackay et al. 2002 [19]	To determine whether there is an effect of practice distribution in the medical setting.	United Kingdom	Randomized control trial	41	Undergraduate and postgraduate students, no previous experience	MIST VR simulator (Mentis, Gothenburg, Sweden)	Spaced 1 (*n*=14): 4 sessions in total (5 min each), with breaks (2,5 min) in between	27,5 min	2,5 min	Laparoscopic transfer-place task: picking an object up with one instrument, transferring it to the other instrument and finally placing in a space on a wire frame.	Time, error, pathlength economy	Post-training: retention test after 5 minutes of rest	Spaced 2 significantly outperformed the massed group (*p* < .05).
							Spaced 2 (*n*=13): 3 sessions in total (5 min each) with breaks (2,5 min) in between	20 min	2,5 min				
							Massed (*n*=14): 1 session (20 min)	20 min	none				
Andersen et al. 2015 [34]	To explore the learning curves of VR simulation training of mastoidectomy and the effects of different practice sequences	Denmark	Prospective cohort study	43	Undergraduate medical students, no previous experience	Visible ear simulator (Freeware, Internet)	Spaced (*n*=21): 6 sessions in total, 1 session with 2 tasks (max 1h) at least 3 days spaced from the next session	> 46 days	≥3 days, mean 7.7 days	Completion of a mastoidectomy with entry into the antrum and posterior tympanotomy	Final product assessment with a 26-item modified welling scale	Assessment of training metrics	Spaced training yielded a significantly higher mean end score compared to massed training (*p* < .01)
							Massed (*n*=19): 1 session in total (with 12 tasks) all in 1 day	1 day	none				
Kang et al. 2015 [35]	To compare different training schedules and identify the most effective.	Korea	Prospective nonrandomized study	30	Surgical novices, no previous experience	The Mimic dV-Trainer (Mimic Technologies, Ic. Seattle, WA)	Spaced 1 (*n*=10): 4 sessions in total, 1 session (1h each) per day for 4 consecutive days	4 days	1 day	Suturing exercise, simulating an anastomosis. The user is required to join 2 adjacent tubes by means of 4 sutures	Time to completion	Assessment of training metrics	Median time to completion was significantly lower in spaced 1 group (daily practice) when compared to group 2 (weekly practice) (*p* < .011). Correlation coefficient calculations of measurements of improvement between each attempt was larger in spaced 1 (daily practice) (-.924) than in spaced 2 (weekly training) (-.899) and the massed group (-.838). The training schedule of spaced 1 was the most effective.
							Spaced 2 (*n*=10): 4 sessions in total, 1 session (1h each) per week for 4 consecutive weeks	4 weeks	7 days				
							Massed (*n*=10): 1 session (4h)	4 hours	none				
Bjerrum et al. 2016 [36]	To compare two distributed practice schedules	Denmark	Randomized control trial	20	Postgraduate medical students, no previous experience	Accutouch (CAE Healthcare, Quebec, Canada)	Spaced 1 (*n*=10): 3 sessions in total, 1 per week for 3 weeks	3 weeks	7 days	Per session training with 3 bronchoscopy simulator cases for a total of 60 minutes.	Procedure time, percent segments entered, wall collisions with the wall obstructing the scope (“red-out”), percent-segments-entered-per-minute	1.Pre-test 2. Assessment after each of the 3 practice sessions 3. Post training: Retention test after 4 weeks	No main effect of group (*p* = n.s.), thus no difference in test scores between one-day spaced training and weekly distributed training.
							Spaced 2 (*n*=10): 3 sessions in total, all in 1 day, spaced by 2 large breaks	1 day	two breaks				
Güldner et al. 2017 [37]	To analyse the effect of differently scheduled training on surgical performance metrics	Germany	Prospective cohort study	40	Novice residents, no previous experience	Da Vinci Surgical Skills Simulator (dVSS; Intuitive Surgical, USA)	Spaced 1 (*n*=20): 15 sessions in total, 1 session on workdays (1-2 tasks each), followed by a 2-day break, repeated for 3 weeks	3 weeks	1 day + 3 days	Exercise 1: pick up rings from a row of pegs and transfer them to another peg. Exercise 2: Pick up 3D objects (wooden letters and number blocks) and set them into corresponding cut-outs. Exercise 3: Draw coloured rings along twisted rods toward the goal of the same colour. Exercise 4: Cauterize and cut dendritic blood vessels, aggravated by rebleeding of the vessels. Exercise 5: Position coloured needles into two colour matched targets of different sizes.	Time to complete, economy of motion, number of instrument collisions, excessive instrument force, instruments out of view, master workspace range, number of drops Overall score (cumulative) Number of missed targets, misapplied energy time, blood loss, and broken vessels	Assessment of training metrics	The spaced group 1 got significantly better overall score, time to complete, and economy of motion in exercise 2 and 3 (*p* < .05). In the exercise 4 and 5, the daily-spaced group 1 performed significantly better with regard to overall score, time to completion, and economy of motion (p < .05).
							Spaced 2 (*n*=20): 3 sessions in total, 1 session (with 6 tasks) per week, repeated after 7 days	3 weeks	7 days				
Gallagher et al. 2012 [38]	To compare the efficacy of 2 identical laparoscopic skill instructions on 2 different training schedules.	Ireland	Randomized control trial	24	Novices with no previous experience	MIST VR simulator (Mentis, Gothenburg, Sweden)	Spaced (*n*=12): 3 sessions in total, 1 session (with 6 tasks) per day, spaced over 3 consecutive days	3 days	1 day	Make as many incisions as possible between evenly spaced (1cm) marks on the long edge of a sheet of paper.	Accuracy of incision	Post training: Completing the cutting task on 5 consecutive days. The massed group was tested on the day of training and the following 4 days. The spaced group was tested on the day of training and the following 2 days	The spaced group had the fastest learning rate overall and on completion of the training outperformed the massed group significantly (*p* < .01).
							Massed (*n*=12): 3 sessions in total (with 6 tasks) over 1 day	1 day	none				
Verdaasdonk et al. 2006 [39]	To determine whether massed or spaced training is the most effective for training endoscopic psychomotor skills.	Netherlands	Randomized control trial	20	Students, no previous experience	SIMENDO VR (Delltatech, Delft, The Netherlands)	Spaced (*n*=10): 3 sessions in total, 1 session (with 4 tasks) per day for 3 consecutive days	3 days	1 day	1. Drop the ball (picking and placing 3 balls in holes) 2. The ring (passing a needle through two rings with both hands) 3. 30° endoscope handling (picking and placing 4 balls on a box with the right hand and an endoscopic camera in the left hand)	Time to completion, collisions of instruments with nontarget environment, pathlength of left and right instrument	Post-training, 7 days after training, identical exercises	The spaced group performed significantly faster (18.7%) than the massed group (*p* < .05). Although the spaced group had fewer collisions and shorter path length for the right instruments and a longer path length for the left instrument, the differences were not significant. The time score differed significantly for exercise 1 (*p* < .05) and exercise 2 (p <.05) between the spaced and the massed group, but not for the endoscope handling exercise.
							Massed (*n*=10): 3 sessions in total, 1 session (with 4 tasks), followed by a break (15 min)	1 day	15 min				

### Is spaced training of psychomotor skills superior to massed training?

Five out of seven studies compared a massed to a spaced training schedule and concluded that spaced training on a VR simulator was superior to massed training. Important to note is that the five studies showed similar findings even though they focused on different psychomotor skills and used non-identical metrics, such as time to completion or economy of motion. Groups with spaced training schedules showed significantly larger performance increase [34], a steeper learning curve [38], were more efficient or completed their training faster or with higher composite scores than massed training groups [19, 35, 37, 39].

### Is there an optimal interval for VR simulator-based psychomotor training sessions?

The studies included in our systematic review varied substantially regarding training schedule, training duration and skills trained. In most studies (*n* = 6), the researchers adopted a weekly training schedule or spaced the training over several consecutive days. The main reason for these spacing rhythms was that weekly or daily training schedules were most compatible with clinical practice [37]. Therefore, predominantly 3 training schedules models were compared: Massed, daily, and weekly training.

A daily training on consecutive days, as adopted by Güldner et al. and Kang et al., resulted in a psychomotor training effect superior to that of a weekly training schedule [35, 37]. Participants in a training programme spaced over consecutive days outperformed participants in a massed, single day training programme of equal duration [38, 39]. Bjerrum et al. did not find any differences in the effectiveness of two spaced training schedules for acquiring bronchoscopy skills [36]. They compared a group with a weekly spaced schedule with a group completing the same training in 3 spaced sessions within 1 day. Hence, from this limited sample, the optimal temporal spacing interval for VR-based psychomotor skill acquisition seems to be daily training.

## Discussion

Overall, we found that spacing of VR simulator training yielded higher performance scores, faster skill acquisition, and improved training metrics compared to a massed training schedule [19, 34, 35, 37, 38]. Such an effect was found across a variety of surgical psychomotor skills and with different spacing intervals. The included studies were too heterogeneous in terms of spacing interval, obtained performance metrics and psychomotor skills trained to allow for a meta-analysis to determine the optimal spacing interval. It seems that interventions with daily training held on consecutive days yield the best outcomes in terms of skill acquisition. This is in line with the outcomes of studies using non-VR simulator training in other healthcare domains, such as weekly vascular anastomosis training, daily endoscopic suturing practice or spaced neonatal intubation training [18, 20, 24, 25].

An explanation for our cautious interpretation –that daily practice of surgical psychomotor skills on a VR simulator may be superior to training with shorter and longer temporal spacing intervals– may lie in an inverted U-curve correlation between spacing interval and skill acquisition, as suggested by Smith et al. [40] Reactive inhibition negatively impacts the effectiveness of massed or distributed training using short (e.g., 15 minutes) inter-training intervals. At the same time, longer inter-training intervals (e.g., 14 days) result in skill deterioration, forgetting and ineffective training [41, 42]. Consequently, the optimal spacing interval for skill acquisition should be long enough to minimize the effect of reactive inhibition and short enough to reduce loss of skill.

Although our outcomes did not allow for a meta-analysis to determine the optimal spacing interval, the question remains whether it is possible to identify one optimal spacing interval for all kinds of surgical psychomotor training. It may be, as Donovan et al. suggested, that the optimal spacing interval is skill dependent due to differences in mental requirements, physical requirements and overall complexity of skills [19, 23, 41]. If so, spacing intervals may need to be tailored to the specific surgical psychomotor skills, and take factors like cognitive and physical demand, overall complexity of skills, level of experience of the trainees and aptitude into consideration. For instance, the cognitive consolidation of complex skills that require more cognitive effort, like orientation and pattern recognition, may require more time for cognitive consolidation [36], and, therefore, longer spacing intervals than simple skills, although the latter may benefit from spaced training as well [23, 40]. Overall, spaced groups have the steepest learning curve and spacing seems to be particularly effective in the early phase of learning a new skill (i.e. “declarative phase”), where the skill is still relatively new and usually many errors occur [27, 38]. Andersen et al. reported that, although the massed training group initially showed faster skills acquisition, their performance started to decline after just 4 repetitions, while the performance of the spaced group asymptotically increased towards a proficiency plateau [34]. With progressive consolidation of skills and a shift into the “procedural phase”, trainees build on their existing experience and their numbers of errors decrease [2, 27, 34].

The multitude of influencing factors does not allow for a single, definite explanation for the spacing effect, but there are some theories that may explain the mechanisms underlying the effectiveness of spaced training. Generally speaking, psychomotor skills are slowly acquired over consecutive training sessions and up to 6 hours after a session [43]. Throughout the training phase, practice provides input for subsequent consolidation of the skill into the respective cognitive region and memory [17, 18, 40, 43]. During subsequent repetitions, the trainee needs to retrieve the memory of the skill required for its execution, which reconsolidates the memory [40]. This reconsolidation also allows additionally acquired movements or information to be integrated into the existing memory [40]. The process of retrieval during successive training sessions is a key aspect of spaced skill acquisition, because each retrieval results in more profound (re)consolidation into the memory [18, 40]. Since spaced training is distributed over repeated educational encounters, active retrieval is required at the beginning of each session. Accordingly, retrieval and reconsolidation are repeatedly executed, and the psychomotor skill is progressively acquired. It is also assumed by Wang et al. that skill consolidation is adaptive, so regular short training intervals signal the brain that the skill will be regularly used in the future [44]. This could stimulate better skill encoding and thereby enhance skill acquisition from repeated learning sessions [44]. Additionally, Spruit et al. attributed the effectiveness of spacing to the beneficial effects of rest and sleep [17]. A trained skill can be negatively influenced by another skill that is trained immediately after training the first skill [20, 43]. In some sense, the memory is overwritten by more recently acquired information. This vulnerability to secondary skills is described as retrograde interference and underlines that consolidation also occurs in a phase immediately after training [43]. Sleep or rest help overcome this effect by providing a period of muted sensory input, thereby enhancing training effectiveness [17]. This aspect makes spaced training so effective: the trainee is provided with a prolonged period of non-practice after a training session and consequently the skill can be consolidated without interference [17]. Therefore, sleep plays a crucial role in the formation, recovery and retention of psychomotor skill memory [20, 21]. Since spaced training often involves at least one night in between two sessions, sleep can positively impact psychomotor skill acquisition. Furthermore, the spacing of training sessions minimizes the aforementioned negative impact of reactive impedance during training [21]. Some authors also hypothesized that longer rest phases in between training sessions allow for supplementary mental practice and rehearsal, often referred to as “reminiscence” [19]. Please note that these are just some theoretical explanations and alleged mechanisms for the effectiveness of spaced psychomotor training. The reality might be more complex and involve multiple mechanisms at the same time.

### Quality of included studies

The included studies were not without methodological limitations. Low numbers of participants and predominant recruitment from a single institution may have introduced bias. In addition, the measured performance metric “time to completion” (i.e., how fast a learner can execute a task/single repetition) may be an accurate measure of skill proficiency but is inadequate as a single performance criterion, as discussed by Kang et al. [35] Learners can execute a task “quick and dirty” (i.e., fast but with limited dexterity, such as messy tool handling), which makes this metric insensitive to safety-compromising tool handling or movements. Instead, it should be used in conjunction with qualitative metrics such as number of errors or excessive instrument force, as described by Güldner et al. [37] After all, the whole idea of VR simulator-based training is that it provides a realistic, consequence-free environment without putting patients at risk so users can learn a skill, perform consistently and reduce errors to a bare minimum [3]. Despite the heterogenicity and limitations of the included studies, the superiority of spaced training has been demonstrated across all studies, which is in line with findings in other healthcare domains [18, 20, 24, 25].

### Strengths and limitations

Strengths of our systematic review were the focus on sensitivity rather than specificity during the search phase and our rigorous methodology, including double-screening substantially more titles and abstracts than the minimum number of 20 to 30 mentioned in best practice guidelines for abstract screening [31], and leniently marking records as “potentially relevant” to foster the identification of relevant studies. Our comprehensive search allowed us to contextualize the current evidence on the spacing of VR-based surgical training with theoretical background articles and findings in other domains. We were able to demonstrate the beneficial effect of spaced training across a variety of settings and skills, which supports the overall validity of our findings. To our knowledge, this is the first systematic review specifically focusing on the spacing of VR simulator-based surgical psychomotor skill acquisition.

We limited our review to the clinical training setting and VR simulators since VR-simulators are not only more advanced than other simulators like box trainers, but also becoming increasingly important in clinical training. This narrow scope is a limitation of the present review since studies about spacing effects in other contexts were not included in our study. Despite the specific focus on VR simulators, there are substantial differences between the different types of VR simulators, for instance in terms of the metrics obtained or the accuracy of their simulation. The limited number of studies included as well as considerable inter-study variability in terms of spacing interval and study design prevented us from conducting a meta-analysis, which may have negatively impacted the overall generalizability of our findings regarding the optimal spacing interval of surgical psychomotor skill training. Nevertheless, our findings do indicate that spacing may improve the effectiveness of VR simulator-based surgical psychomotor training, which may in turn support the validity of our conclusions.

### Implications for future research and practice

To further clarify the effect of spacing on psychomotor skill acquisition, future research should focus on specific skills and other training modalities (e.g., non-VR trainers). For instance, a large-scale multicentre study comparing weekly and daily VR simulator-based surgical psychomotor training may help determine the optimal spacing interval for a variety of surgical motor skills. Despite its limitations, our review showed that the training of novice doctors can be further improved. By practicing on VR simulators, novice doctors can reach a minimum proficiency level in a safe, virtual environment before applying the trained skill in the operating room, thereby increasing quality of care without sacrificing patient safety. Given the faster skill acquisition when using a spaced training schedule, surgical trainees can reach proficiency in a new surgical skill in a shorter total training time. Nevertheless, some trainees may need more time or more repetitions to reach proficiency in a new skill [12, 45]. Fortunately, a spaced curriculum can be adjusted to individual schedules and learning curves [3]. Considering patient safety, operating room efficacy and costs, it would be reasonable to abandon the traditional Halstedian approach and its shortcomings, and instead implement VR simulator-based training using a spaced training schedule [46]. A potential barrier to the implementation of spaced training may be the busy schedules of junior residents in training. Given the benefits of spaced training, further research is needed to identify enablers and barriers to its implementation and develop strategies to support the implementation of spaced VR-training.

## Conclusion

Our systematic review showed that spacing is superior to massed training and is beneficial for surgical psychomotor skill training on VR simulators. Spaced training resulted in better performance scores and faster skill acquisition than massed training. Based on the heterogeneous sample of seven included studies, the optimal spacing interval for VR-based psychomotor training seems to be daily practice on consecutive days. Since the studies included in our review were too heterogeneous to allow for a meta-analysis to substantiate this interpretation, additional large-scale cohort studies are needed to confirm this optimal spacing interval. Furthermore, future research should focus on identifying optimal spacing intervals by comparing the acquisition of surgical psychomotor skills using daily training on consecutive days, weekly training, or even training with incrementally increasing training intervals, taking into account factors potentially influencing the effectiveness of different training intervals, like overall complexity of skills, cognitive and physical aptitude as well as previous experience of the trainee.

## Supplementary Information

**Additional file 1: Supplementary file 1.** Search String. An exemplary overview of the search string used during the review process.

**Additional file 2: Supplementary file 2.** PRISMA Flow diagram. Full PRISMA 2020 flow diagram.

**Additional file 3: Supplementary file 3.** MERSQI scores of the included studies.

**Additional file 4: Supplementary file 4.** Risk of Bias Assessment of the included studies including substantiation of the assessments.

## Data Availability

All data analysed in this review study are referred to in this published article and its supplementary information files.
