# Supplementary material for: Towards best practice in developing motor skills: a systematic review on spacing in VR simulator-based psychomotor training for surgical novices
Source: BMC Med Educ. 2023 Mar 13;23:154. doi: 10.1186/s12909-023-04046-1 (PMC10009969; doi:10.1186/s12909-023-04046-1)
Supplement: Supplementary file 1 — Additional file 1: Supplementary file 1. Search String. An exemplary overview of the search string used during the review process. [file 12909_2023_4046_MOESM1_ESM.docx]

# Supplementary file 1: Search string

PubMed

| S6 | S1 AND S2 AND S3 AND S4 AND S5 |
| --- | --- |
| S5 | “Augmented reality”[Title/Abstract] OR ar[Title/Abstract] OR virtual[Title/Abstract] OR VR[Title/Abstract] OR simulat*[Title/Abstract] OR “box trainer”[Title/Abstract] OR “skill lab”[Title/Abstract] OR “skill labs”[Title/Abstract] OR “skill laboratory”[Title/Abstract] OR “skill laboratories”[Title/Abstract] OR “skill training”[Title/Abstract] OR “skills lab”[Title/Abstract] OR “skills labs”[Title/Abstract] OR “skills laboratory”[Title/Abstract] OR “skills laboratories”[Title/Abstract] OR “skills training”[Title/Abstract] |
| S4 | novice OR trainee* OR “medical student” OR “medical students” OR “clinical student” OR “clinical students” OR residen* OR internship* OR clerkship* OR HPE OR “health professions education” OR “medical school” OR “med school” OR “medical education” OR “medical training” OR “healthcare” OR “postgraduate training” OR “postgraduate education” OR “postgraduate medical education” OR “clinical training” OR “clinical education” OR CME OR PGME OR Surgical OR (MH "Education, Medical+") OR (MH "Education, Medical, Undergraduate") OR (MH "Education, Medical, Graduate+") OR (MH "Education, Medical, Continuing") OR (MH “Schools, Medical”) OR (MH " Students, Medical”) OR (MH “Internship and Residency”) |
| S3 | dexter*[Title/Abstract] OR motor*[Title/Abstract] OR psychomotor*[Title/Abstract] OR skill*[Title/Abstract] OR Movement[Title/Abstract] OR Motion*[Title/Abstract] OR Stitch*[Title/Abstract] OR Grasp*[Title/Abstract] OR Laparoscop*[Title/Abstract] OR Thoracoscop*[Title/Abstract] OR Arthroscop*[Title/Abstract] OR Endoscop*[Title/Abstract] |
| S2 | “Distributed learning”[Title/Abstract] OR “Distributed teaching”[Title/Abstract] OR “Distributed education”[Title/Abstract] OR “Distributed practice”[Title/Abstract] OR “Distributed training”[Title/Abstract] OR “Distributed modules”[Title/Abstract] OR “Distributed modelling”[Title/Abstract] OR “training distribution”[Title/Abstract] OR “Practice distribution“OR “module distribution”[Title/Abstract] OR Massed[Title/Abstract] OR Spac*[Title/Abstract] OR interval[Title/Abstract] |
| S1 | OSATS OR Complet* OR duration OR period OR span OR Error OR “Motion economy” OR “Movement economy” OR “Motion efficiency” OR “Movement efficiency” OR Perform* OR Succe* OR Competen* OR “Skill decay” OR proficien* OR Acqui* OR Retention OR Retriev* OR Consolidat* OR Translat* OR Transfer* OR Effect* OR Evaluat* OR Assess* OR Rati* OR Rate* OR Scor* OR Judg* OR Apprais* OR Grade* OR Mark* OR Improv* OR Enhanc* OR Progress* OR Advanc* OR Develop* OR Growth OR Increas* OR ("task performance and analysis"[MeSH Terms]) OR ("retention, psychology"[MeSH Terms]) OR („Memory“[MeSH Terms]) OR („Learning“[MeSH Terms]) OR ("transfer, psychology"[MeSH Terms]) |
