# Supplementary material for: Towards best practice in developing motor skills: a systematic review on spacing in VR simulator-based psychomotor training for surgical novices
Source: BMC Med Educ. 2023 Mar 13;23:154. doi: 10.1186/s12909-023-04046-1 (PMC10009969; doi:10.1186/s12909-023-04046-1)
Supplement: Supplementary file 2 — Additional file 2: Supplementary file 2. PRISMA Flow diagram. Full PRISMA 2020 flow diagram. [file 12909_2023_4046_MOESM2_ESM.docx]

Reports assessed for eligibility

not applicable

Reports excluded:

not applicable

**Identification of studies via other methods**

Reports sought for retrieval

not applicable

Reports not retrieved

not applicable

**Identification of studies via databases and registers**

Records identified from:

Websites (n = 0)

Organisations (n = 0)

Reference lists (n = 0)

Citation searching (n = 0)

Records removed *before screening*:

Duplicate records removed (n = 197)

Records (n=1859) identified from:

ERIC (n = 168)

CINAHL (n = 333)

PubMed (n = 1161)

PsycINFO (n = 145)

Psychology and Behavior Sciences Collection (n = 52)

**Identification**

Records excluded

(n = 1609)

Records screened

(n = 1662)

Reports sought for retrieval

(n = 53)

Reports not retrieved

(n = 0)

**Screening**

Reports excluded: 46

Reason 1: not using a VR simulator

Reason 2: published in a non- peer-reviewed journal

Reason 3: not a primary study focused on the effect of spacing on skills acquisition

Reports assessed for eligibility

(n = 53)

Studies included in review

(n = 7)

**Included**

*Consider, if feasible to do so, reporting the number of records identified from each database or register searched (rather than the total number across all databases/registers).

**If automation tools were used, indicate how many records were excluded by a human and how many were excluded by automation tools.

*From:*  Page MJ, McKenzie JE, Bossuyt PM, Boutron I, Hoffmann TC, Mulrow CD, et al. The PRISMA 2020 statement: an updated guideline for reporting systematic reviews. BMJ 2021;372:n71. doi: 10.1136/bmj.n71. For more information, visit: <http://www.prisma-statement.org/>
