# Supplementary material for: Towards best practice in developing motor skills: a systematic review on spacing in VR simulator-based psychomotor training for surgical novices
Source: BMC Med Educ. 2023 Mar 13;23:154. doi: 10.1186/s12909-023-04046-1 (PMC10009969; doi:10.1186/s12909-023-04046-1)
Supplement: Supplementary file 3 — Additional file 3: Supplementary file 3. MERSQI scores of the included studies. [file 12909_2023_4046_MOESM3_ESM.docx]

# Supplementary file 3: MERSQI scores of the included studies

| **Study design** | **Score** | Mackay et al. 2002 [19] | Andersen et al. 2015 [34] | Kang et al. 2 2015 [35] | Bjerrum et al. 2016 [36] | Güldner et al. 2017 [37] | Gallagher et al. 2012 [38] | Verdaasdonk et al. 2006 [39] |
| --- | --- | --- | --- | --- | --- | --- | --- | --- |
| single- group cross sectional or single- group post-test only | **1** |  |  |  |  |  |  |  |
| Single-group pre-test and post-test | **1,5** |  |  |  |  |  |  |  |
| Nonrandomized, 2 group | **2** |  | 2 | 2 |  | 2 |  |  |
| RCT | **3** | 3 |  |  | 3 |  | 3 | 3 |
|  |  |  |  |  |  |  |  |  |
| **Sampling: number of institutions studied** |  |  |  |  |  |  |  |  |
| 1 institution | **0,5** | 0,5 | 0,5 | 0,5 | 0,5 | 0,5 | 0,5 |  |
| 2 institutions | **1** |  |  |  |  |  |  | 1 |
| ≥3 institutions | **1,5** |  |  |  |  |  |  |  |
|  |  |  |  |  |  |  |  |  |
| **Sampling: response rate** |  |  |  |  |  |  |  |  |
| n.a. | **0** |  |  |  |  |  |  |  |
| <50% or not reported | **0,5** |  |  |  |  |  |  |  |
| 50%-74% | **1** |  |  |  |  |  |  |  |
| ≥75% | **1,5** | 1,5 | 1,5 | 1,5 | 1,5 | 1,5 | 1,5 | 1,5 |
|  |  |  |  |  |  |  |  |  |
| **Type of data** |  |  |  |  |  |  |  |  |
| Assessment by study participants | **1** |  |  |  |  |  |  |  |
| Objective measurement | **3** | 3 | 3 | 3 | 3 | 3 | 3 | 3 |
|  |  |  |  |  |  |  |  |  |
| **Validity of evidence for evaluation instrument scores** |  |  |  |  |  |  |  |  |
| n.a. | **0** | n.a. | 0 | 0 | 0 | 0 | 0 | 0 |
| Internal structure | **1** | 1 | n.a. | n.a. | n.a. | n.a. | n.a. | n.a. |
| Content | **1** | 1 | 1 | 1 | 1 | 1 | 1 | 1 |
| Relationships to other variables | **1** | 1 | 1 | 1 | 1 | 1 | n.a. | 1 |
|  |  |  |  |  |  |  |  |  |
| **Data analysis appropriateness** |  |  |  |  |  |  |  |  |
| statistical error of data not properly analysed at all | **0** |  |  |  |  |  |  |  |
| Data analysis appropriate for study design | **1** | 1 | 1 | 1 | 1 | 1 | 1 | 1 |
|  |  |  |  |  |  |  |  |  |
| **Data analysis sophistication** |  |  |  |  |  |  |  |  |
| descriptive analysis only | **1** |  |  |  |  |  |  |  |
| beyond descriptive analysis | **2** | 2 | 2 | 2 | 2 | 2 | 2 | 2 |
|  |  |  |  |  |  |  |  |  |
| **Outcome** |  |  |  |  |  |  |  |  |
| satisfaction, attitudes, perception, general facts | **1** |  |  |  |  |  |  |  |
| Knowledge, skill | **1,5** | 1,5 | 1,5 | 1,5 | 1,5 | 1,5 | 1,5 | 1,5 |
| behaviour | **2** |  |  |  |  |  |  |  |
| patient/health care outcome | **3** |  |  |  |  |  |  |  |
|  |  |  |  |  |  |  |  |  |
| Total MERSQI score | **18** | 16 | 14 | 14 | 15 | 14 | 14 | 15 |
| Mean | **14,14** |  |  |  |  |  |  |  |
