# Supplementary material for: Towards best practice in developing motor skills: a systematic review on spacing in VR simulator-based psychomotor training for surgical novices
Source: BMC Med Educ. 2023 Mar 13;23:154. doi: 10.1186/s12909-023-04046-1 (PMC10009969; doi:10.1186/s12909-023-04046-1)
Supplement: Supplementary file 4 — Additional file 4: Supplementary file 4. Risk of Bias Assessment of the included studies including substantiation of the assessments. [file 12909_2023_4046_MOESM4_ESM.docx]

# Supplementary file 4: Risk of Bias Assessment of the included studies including substantiation of the assessments.

Mackay et al. 2002 [19]

| **Bias** | **Authors judgement** | **Support for Judgement** |
| --- | --- | --- |
| Random sequence generation (selection bias) | Low risk | Comment: "Subjects were randomly allocated to one of three training regimes." |
| Allocation concealment (selection bias) | Low risk | Comment: "Subjects were randomly allocated to one of three training regimes." |
| Blinding of participants and personnel (performance bias) | Low risk | Comment: No blinding due to study design but unlikely to influence study outcome. |
| Blinding of outcome assessment (detection bias) | Low risk | Comment: No blinding, but outcome is unlikely to be influenced. |
| Incomplete outcome data (attrition bias) | Low risk | Comment: No apparent loss of data or incomplete data. |
| Selective reporting (reporting bias) | Low risk | Comment: Protocol not available but all pre-specified outcomes were reported for each individual exercise. "It was planned that analysis would be on the basis of overall score and the three major components thereof: time, errors. and path length economy." |
| Other bias | Unclear risk | None were identified. |

Andersen et al. 2015 [34]

| **Bias** | **Authors judgement** | **Support for Judgement** |
| --- | --- | --- |
| Random sequence generation (selection bias) | High risk | Comment: Recruiting of students from different semesters without previous experience. All participants were recruited from a single institution. Randomization of students "...not possible for practical reasons". |
| Allocation concealment (selection bias) | High risk | Comment: The allocation of participants to distributed or massed group was not described in detail. There were differences in characteristics between 2 practice groups regarding gender and video gaming frequency. |
| Blinding of participants and personnel (performance bias) | Low risk | Comment: No blinding, since participants were randomly assigned to two different practice schedules. Unlikely to influence study outcome. |
| Blinding of outcome assessment (detection bias) | Low risk | The virtual temporal bone final products that were autosaved at the end of the 30-minute sessions were assessed by 2 expert raters (P.C.-T. and M.S.S.) who were blinded to participant, session number, and practice and tutoring groups (examples of final product progression are shown in Figure 2). The objective, structured assessments (OSATS) developed for bronchoscopy simulation were deselected due to the lack of opportunity for blinding of the assessors (18) and due to lack of feasibility (19). |
| Incomplete outcome data (attrition bias) | High risk | Comment: 3 participants out of distributed group did not complete sessions, without stated reasons. The missing data is not balanced across groups. |
| Selective reporting (reporting bias) | Unclear risk | Comment: Performance of training reflected by 26-item modified Welling Scale for final product analysis |
| Other bias | Low risk | None were identified. |

Kang et al. 2015 [35]

| **Bias** | **Authors judgement** | **Support for Judgement** |
| --- | --- | --- |
| Random sequence generation (selection bias) | High risk | Comment: "A total of 30 medical students were enrolled in this prospective nonrandomized, institutional review board–approved study. [...] This study was not performed as randomized trial. " |
| Allocation concealment (selection bias) | High risk | Comment: "A total of 30 medical students were enrolled in this prospective nonrandomized, institutional review board–approved study." |
| Blinding of participants and personnel (performance bias) | Low risk | Comment: No blinding due to study design but unlikely to influence study outcome. |
| Blinding of outcome assessment (detection bias) | Low risk | Comment: No blinding, but outcome is unlikely to be influenced. "The total task time in the simulator was recorded automatically using a computerized algorithm created by the manufacturer." |
| Incomplete outcome data (attrition bias) | Low risk | Comment: No apparent loss of data or incomplete data. |
| Selective reporting (reporting bias) | Low risk | Comment: All outcome data for all groups were reported. |
| Other bias | Unclear risk | None were identified. |

Bjerrum et al. 2016 [36]

| **Bias** | **Authors judgement** | **Support for Judgement** |
| --- | --- | --- |
| Random sequence generation (selection bias) | Low risk | Comment: "Each participant [...] was randomly assigned to one-day distributed practice or weekly distributed practice using a randomisation procedure with closed envelopes." |
| Allocation concealment (selection bias) | Low risk | Comment: "Each participant [...] was randomly assigned to one-day distributed practice or weekly distributed practice using a randomisation procedure with closed envelopes." |
| Blinding of participants and personnel (performance bias) | Low risk | Comment: No blinding, since participants were randomly assigned to two different practice schedules. Unlikely to influence study outcome. |
| Blinding of outcome assessment (detection bias) | Low risk | Comment: "Participants were tested with bronchoscopy simulator metrics. These included ‘procedure time’, ‘percent-segments-entered’, ‘wall collisions’, and ‘red-out’. The combined measure ‘percent-segments-entered-per-minute’ was chosen as the primary outcome measure [...]. The objective, structured assessments (OSATS) developed for bronchoscopy simulation were deselected due to the lack of opportunity for blinding of the assessors and due to lack of feasibility. |
| Incomplete outcome data (attrition bias) | Low risk | Comment: No missing data reported. |
| Selective reporting (reporting bias) | Low risk | Comment: Protocol not available but test data for all measures were reported as outlined in the pre-specified outcomes of interest. |
| Other bias | Unclear risk | None were identified. |

Güldner et al. 2017 [37]

| **Bias** | **Authors judgement** | **Support for Judgement** |
| --- | --- | --- |
| Random sequence generation (selection bias) | Low risk | Comment: "A total of 40 participants, residents in their first years, were included and randomly distributed among two equal groups, which differed in their training interval." |
| Allocation concealment (selection bias) | Low risk | Comment: "A total of 40 participants, residents in their first years, were included and randomly distributed among two equal groups, which differed in their training interval." |
| Blinding of participants and personnel (performance bias) | Low risk | Comment: No blinding due to study design but unlikely to influence study outcome. |
| Blinding of outcome assessment (detection bias) | Low risk | Comment: No blinding, but measurement unlikely to be influenced, because metrics were obtained by the simulator. "Assessed parameters were time to complete the exercise, economy of motion, number of instrument collisions, excessive instrument force, instruments out of view, master workspace range, number of drops, and the overall score, which combined all previous metrics. [...] as well. For the two final exercises, additional parameters were also recorded (“Needle Targeting”: the number of missed targets; “Energy Dissection 2”: misapplied energy time, blood loss, and broken vessels)." |
| Incomplete outcome data (attrition bias) | Low risk | Comment: No loss of data or incomplete data reported. |
| Selective reporting (reporting bias) | Low risk | Comment: Protocol not available but all pre-specified outcomes were reported for each individual exercise. |
| Other bias | Unclear risk | None were identified. |

Gallagher et al. 2012 [38]

| **Bias** | **Authors judgement** | **Support for Judgement** |
| --- | --- | --- |
| Random sequence generation (selection bias) | Low risk | Comment: "All subjects were randomized to 1 of 3 experimental conditions. Numbers 1 to 24 were assigned to 1 of 3 equal size groups using a research number randomizer." |
| Allocation concealment (selection bias) | Low risk | Comment: "Each number and their group allocation were kept in an envelope and retrieved (from A.G.G.) just before subject participation in the study." |
| Blinding of participants and personnel (performance bias) | Low risk | Comment: No blinding due to study design but unlikely to influence study outcome. |
| Blinding of outcome assessment (detection bias) | Low risk | Comment: "The cutting task performance assessor was blinded as to subject group." |
| Incomplete outcome data (attrition bias) | Low risk | Comment: All outcome data for all groups were reported. |
| Selective reporting (reporting bias) | Low risk | Comment: Protocol not available but all pre-specified outcomes were reported. |
| Other bias | Unclear risk | None were identified. |

Verdaasdonk et al. 2007 [39]

| **Bias** | **Authors judgement** | **Support for Judgement** |
| --- | --- | --- |
| Random sequence generation (selection bias) | Low risk | Comment: "These students were randomly assigned to two groups of 10 subjects each: group A (training over several days) and group B (training on 1 day with short breaks)." |
| Allocation concealment (selection bias) | Low risk | Comment: "These students were randomly assigned to two groups of 10 subjects each: group A (training over several days) and group B (training on 1 day with short breaks)." |
| Blinding of participants and personnel (performance bias) | Low risk | Comment: No blinding due to study design but unlikely to influence study outcome. |
| Blinding of outcome assessment (detection bias) | Low risk | Comment: No blinding, but outcome is unlikely to be influenced.  "The parameters measured were time to completion of the task (seconds), collisions of instruments with the non-target environment (n), and the path length of the right and left instrument (arbitrary units). The parameter scores for all the tasks were summed for each repetition and analysed. Subsequently, the parameter scores also were analysed for each task separately per repetition and compared between the two groups." |
| Incomplete outcome data (attrition bias) | Low risk | Comment: No apparent loss of data or incomplete data.  "Table 2 presents the total scores for the first training cycle, the end of the training, and the post-test." |
| Selective reporting (reporting bias) | Low risk | Comment: All outcome data for all groups were reported. "Table 2". |
| Other bias | Unclear risk | None were identified. |
